# Neurokinin 3 Receptor Antagonists Do Not Increase FSH or Estradiol Secretion in Menopausal Women

**DOI:** 10.1210/jendso/bvz009

**Published:** 2019-11-14

**Authors:** Julia K Prague, Ali Abbara, Alexander N Comninos, Channa N Jayasena, Claire E Higham, Jo Adaway, Brian G Keevil, Johannes D Veldhuis, Waljit S Dhillo

**Affiliations:** 1 Section of Endocrinology & Investigative Medicine, Imperial College, London, UK; 2 Department of Endocrinology, The Christie NHS Foundation Trust, University of Manchester, Manchester Academic Health Science Centre, Manchester, UK; 3 Biochemistry Department, Wythenshawe Hospital, Wythenshawe, UK; 4 School of Medical Sciences, Faculty of Biology, Medicine and Health, University of Manchester, Manchester Academic Health Science Centre, Manchester, UK; 5 Mayo Clinic, Rochester, MN, UK

**Keywords:** NK3R antagonists, breast cancer, estradiol, menopause, flashes

## Abstract

**Background:**

Neurokinin 3 receptor (NK3R) antagonism is a promising novel treatment for menopausal flashes. However, to avoid adverse hormonal effects it is clinically important to first confirm whether gonadotropin and estradiol concentrations change as a result of their administration.

**Methods:**

Single-center, randomized, double-blind, placebo-controlled, crossover trial of an oral NK3R antagonist (MLE4901) in 28 women aged 40 to 62 years, experiencing >7 hot flashes/24 h; some bothersome or severe (Clinicaltrials.gov, NCT02668185). Weekly serum gonadotropins and estradiol levels were measured using commercially available automated immunoassays a priori. Serum estradiol was also measured post hoc using a highly sensitive direct assay by liquid chromatography tandem mass spectrometry. Hormone levels were compared by the paired sample *t* tests or by the Wilcoxon matched-pairs signed rank test, as appropriate for the distribution of the data.

**Results:**

Mean (standard deviation) serum follicle-stimulating hormone (FSH) concentration was not significantly increased when taking MLE4901 (72.07 ± 19.81 IU/L) compared to placebo (70.03 ± 19.56 IU/L), *P* = .26. Serum estradiol was also not significantly altered, irrespective of which assay method was used (median interquartile range of serum estradiol by immunoassay: placebo 36 ± 3 pmol/L, MLE4901 36 ± 1 pmol/L, *P* = .21; median serum highly sensitive estradiol: placebo 12 ± 16 pmol/L, MLE4901 13 ± 15 pmol/L, *P* = .70). However, mean (standard deviation) serum luteinizing hormone concentration significantly decreased with MLE4901 (27.63 ± 9.76 IU/L) compared to placebo (30.26 ± 9.75 IU/L), *P* = .0024.

**Implication:**

NK3R antagonists do not increase serum estradiol or FSH despite their reduction in hot flashes. This is clinically significant and highly reassuring for women who have a contraindication to conventional hormone therapy such as prior/existing breast cancer and/or thromboembolism.

Seventy percent of menopausal women are affected by hot flashes (1), and 10% describe them as intolerable. (2) Symptoms of the menopause are a result of estrogen deficiency and are typically highly disruptive, persist for many years, (3) and affect all aspects of daily life. Hot flashes are reported to be the most bothersome of all menopausal symptoms.(4) Hormone therapy is effective but is not without risk (5) and is contraindicated in some women including those with a history of breast cancer (the most common cancer in women) (6) or thromboembolic disease. Alternative therapies that are currently available such as selective serotonin reuptake inhibitors (some of which are also contraindicated in breast cancer patients), venlafaxine, gabapentin, and herbal remedies such as red clover are not without side effects and/or have variable efficacy or are not widely available such as cognitive behavioral therapy. An effective, safe novel therapy could therefore benefit millions of women worldwide and particularly those women in whom hormone therapy is contraindicated.

Over the last 20 years, studies exploiting animal and human models of hypothalamic neuropeptide signaling have implicated neurokinin B (NKB) together with its receptor (NK3R) in the etiology of menopausal flashes.(7–10) Most recently, data from the first in human, proof of concept, randomized, placebo-controlled study clearly demonstrated that an oral NK3R antagonist can effectively attenuate menopausal flashes.(11) Subsequent industry-led studies of other chemically distinct NK3R antagonists have suggested similar results, including larger scale clinical trials of longer duration with efficacy, safety, and optimal dosing strategy, although the results have not yet been published in full. (12) Thus, oral NK3R antagonists have great promise as novel therapeutics to change future clinical practice.(13)

However, if NK3R antagonists are to become the future treatment for hot flashes in menopausal women and, in particular, in women with a prior history of hormonally receptive breast cancer and/or thromboembolism, then their clinical safety must be established. First, it is essential to confirm that estradiol secretion does not increase with administration of an oral NK3R antagonist. This is critical because although ovarian reserve is limited in the menopause; NK3R antagonists affect LH pulsatility by increasing the amplitude and orderliness of luteinizing hormone (LH) pulses,(11) which could theoretically increase estradiol release from the ovaries, peripheral aromatase activity is increased,(14); and the onset of hot flashes typically occurs when estradiol levels are low but still sufficient to significantly increase the risk of breast cancer progression/recurrence.(15) Furthermore, NKB-secreting neurons express the estradiol receptor, and aromatase is also expressed in a number of regions within the central nervous system.(16) It is therefore plausible that NK3R antagonists could stimulate the release of estradiol from the ovaries in some menopausal women and/or alter local inhibition of estradiol synthesis at the neuronal level with potential safety risks for those with current or prior hormonally receptive breast cancer and/or thromboembolism. Furthermore, serum estradiol measurements are usually below the lower limit of detection in most commercially available estradiol immunoassays making it difficult to monitor. However, serum estradiol measured by higher sensitivity methods such as liquid chromatography tandem mass spectrometry (LCMS) demonstrate detectable and variable estradiol levels in menopausal and postmenopausal women and those on estrogen deprivation therapy.(17) Therefore, if NK3R antagonists could be shown to not increase estradiol levels on high sensitivity assays, this would suggest that they could be used safely in women with current, prior, or high future risk of hormonally receptive breast cancer who have disabling hot flashes due to (i) ovarian toxicity secondary to chemotherapeutic agents; (ii) estrogen deprivation therapy, such as tamoxifen/aromatase inhibitors, to prevent progression/recurrence by minimizing receptor activation/peripheral conversion respectively; and/or (iii) diagnosis after the natural menopause. Second, it is also possible that NK3R antagonists could stimulate the release of follicle-stimulating hormone (FSH) from the anterior pituitary via the gonadotropin-releasing hormone (GnRH) pulse generator. Study of individuals with TAC3 and TACR3 mutations that encode NKB and NK3R has shown they have hypogonadotropic hypogonadism with higher mean FSH:LH ratio secondary to low GnRH pulsatile frequency, which can be normalized by administration of pulsatile GnRH.(18) This could be clinically important as recent animal studies have suggested that high circulating FSH may have detrimental metabolic effects on adipose tissue and bone density, which are commonly seen in the menopause.(19) As such it would be essential to also confirm that FSH secretion does not increase with administration of an oral NK3R antagonist.

In this study, we used a modern, commercially available, automated immunoassay as the current clinical gold standard, as well as a validated highly sensitive estradiol direct assay by LCMS to determine if oral NK3R antagonists alter FSH or estradiol levels.

## Materials and Methods

### Study approvals

Approvals were awarded by the West London Regional Ethics Committee (15/LO/1481), and the Medicine and Healthcare Products Regulatory Agency (EudraCT 2015-001553-32). The trial was registered in full at ClinicalTrials.gov prior to study start (NCT02668185) and performed in accordance with Good Clinical Practice Guidelines.

### Protocol

Women aged 40 to 62 years, who had not had a menstrual period for at least 12 months and who were experiencing at least 7 hot flashes/24 h period, of which some were bothersome or severe, were recruited for this randomized, double-blind, placebo-controlled, single-center, crossover study (Clinicaltrials.gov, NCT02668185). Sixty-eight women were screened of whom 45 were confirmed eligible to enter the initial run-in phase of the study. Thirty-seven women were subsequently confirmed to be eligible to enter the active phase of the study receiving four weeks treatment with an oral selective NK3R antagonist twice daily (MLE4901, Millendo Therapeutics, Inc., Ann Arbor, MI, US) and 4 weeks of exact-match placebo twice daily in random order separated by a 2-week washout period.(11) Twenty-eight women completed the protocol.(11) Full details outlining inclusion and exclusion criteria and study design are as previously described.(11)

Analysis of weekly serum gonadotropin (LH and FSH) and estradiol concentrations (14 weeks in total) were included a priori in our protocol as defined secondary outcomes for independent analysis (NCT02668185) and to ensure that all outcomes were adjusted for serum estradiol level throughout the study period, which could confound occurrence of hot flashes. As such, at each study visit during the 14-week protocol, 3 mL of blood was collected in a plain Vacutainer tube containing no additive (Vacutainer, GP Supplies, UK) from a venipuncture site in the arm and left to clot for at least 30 min. Clotted samples were then centrifuged at 503 rcf for 10 minutes, after which the serum supernatant was removed and frozen at –20°C. Samples were subsequently processed in batches after participants had finished the study protocol to eliminate any intra-assay variation. A modern, commercially available, automated chemiluminescent immunoassay method (Abbott Diagnostics, Maidenhead, UK) was used for analysis as per routine clinical practice (20–22). The respective intra-assay and inter-assay coefficients of variation for each assay were 4.1% and 2.7% (LH), 4.1% and 3.0% (FSH), and 3.3% and 3.0% (estradiol). Analytical sensitivities were 0.5 IU/L (LH), 0.05 IU/L (FSH), and 37 pmol/L (estradiol). Furthermore, to analyze FSH pulsatility, a subgroup of participants based on their willingness to participate (*n* = 13) attended our clinical research facility for two 8-hour studies during the second week of both treatment periods. During both visits, a 3 mL blood sample was taken every 10 minutes from a peripheral venous cannula sited before the study commenced (time: minus 30 minutes [-30 mins]). All blood samples were processed and analyzed as previously outlined for the weekly samples. FSH pulsatility was determined using a blinded deconvolution method with 93% sensitivity and specificity by calculating the number and amplitude of FSH pulses and how ordered they were. (23)

Our ethical permissions allowed retention of any remaining serum after our a priori measurements had been completed for later analysis; 36 of the 37 women who entered the active stage of the study consented to this for each of their weekly samples. As such independent, single-blinded, post-hoc analysis using a highly sensitive estradiol direct assay by LCMS was performed in 36 participants. The assay was performed as previously described. (17) Briefly, samples were anonymized (both for participant and study intervention) and delivered on dry ice to an independent collaborator. Samples were thawed and then supported liquid extraction was performed on 250 µl of each sample using methyl tertiary butyl ether. The extract was dried and reconstituted with 100 µl of 40% methanol. Online automated solid-phase extraction was performed on 75µl of extract using C18 cartridges on a Waters Online Solid-Phase Extraction Manager coupled to a Waters triple quadrupole mass spectrometer. The lower limit for quantification for estradiol was 10 pmol/L. The coefficient of variation of the assay for estradiol concentration of 125 pmol/L was <7% and 10% at 22 pmol/L. The average recovery for estradiol was 102%. The run time was 4.5 min per sample. Once the anonymized results had been returned, unblinding occurred for analysis.

### Statistical analysis

Mean value for each of the 4-week intervention periods (placebo and MLE4901) was calculated for each outcome. As such, analysis was only completed for the per-protocol (*n* = 28) rather than the intention-to-treat (*n* = 37) cohort. This ensured that paired results were comparable in a complete data set to ensure a small effect was not under- or overreported due to missing data. Parametrically distributed data were compared by the paired sample *t* test (FSH, LH), whereas nonparametrically distributed data were compared by the Wilcoxon matched-pairs signed rank test (immunoassay and highly sensitive estradiol). All tests were 2-tailed, and *P* < .05 regarded as statistically significant. As one participant did not consent to further analysis of her remaining serum, 27 participants were included in the analysis of highly sensitive estradiol, whereas for LH, FSH, and estradiol by immunoassay all 28 possible participants were included as these were defined secondary outcomes of the incident trial.

The number of FSH pulses were compared by Poisson regression, and a generalized linear mixed model was used to analyze FSH pulse amplitude and orderliness. Models implemented a standard crossover analysis, with administration sequence and treatment as fixed effects and subject as a random effect.

### Funding

This was an academic investigator initiated and led study, funded by the UK Medical Research Council (grant reference MR/M024954/1) and an NIHR Research Professorship to WSD (grant reference RP-2014-05-001).

## Results

As previously reported, the cohort of 28 participants had a mean age of 55 years (range 49–62 years) and a mean body mass index of 25.8 kg/m^2^ (range 17.9–36.7 kg/m^2^), 74% were white (*n* = 21), and 5 were current smokers.(11) The mean time since onset of hot flashes was 78 months (12–192 months), and the mean time since last menstrual period was 99 months (12–360 months). Four women had previously had a hysterectomy, and 1 woman had previously had a hysterectomy plus bilateral salpingo-oophorectomy.(11) At the screening appointment, mean LH was 32.7 IU/L (16.3–53.0), mean FSH was 73.7 IU/L (34.9–131.0), and for all participants, estradiol was reported as being <70 pmol/L by the performing laboratory.(11) All possible baseline covariates, including duration of amenorrhea and of hot flashes, were included in exploratory analyses of our primary, and all secondary outcomes as per our a priori statistical plan but were removed from the final analysis models when shown not to be significant.

**Figure 1.**
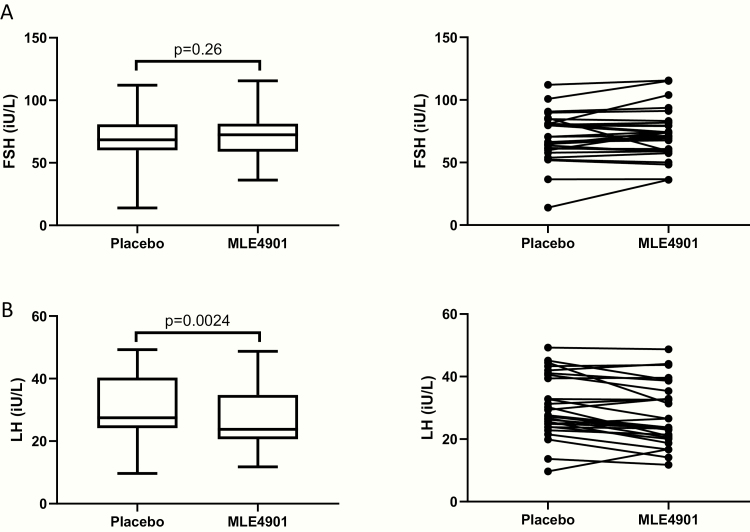
Mean serum gonadotropin concentrations during intervention periods. Gonadotropins were measured weekly using a modern, commercially available, automated chemiluminescent immunoassay method (Abbott Diagnostics, Maidenhead, UK) (20, 21) from a serum sample obtained by peripheral venipuncture as per routine clinical practice. Mean concentration was calculated for the 4 weeks of the intervention periods (placebo and oral neurokinin 3 receptor antagonist [MLE4901]). Statistical analysis used paired *t* tests as the data from the 28 participants who completed the protocol was normally distributed. Tests were 2-tailed, significance was set at *P* < .05. (A) Left panel: follicle stimulating hormone (FSH) serum concentration; box plots: line, median; box, interquartile range; whiskers extend to the extremes of the data (minimum and maximum values). Right panel: individual values of FSH serum concentration. (B) Left panel: LH serum concentration; box plots: line, median; box, interquartile range; whiskers extend to the extremes of the data (minimum and maximum values). Right panel: individual values of LH serum concentration. Reference ranges were LH 4–14 IU/L and FSH 1.5–8 IU/L. The respective intra-assay and inter-assay coefficients of variation for each assay were as follows: 4.1% and 2.7% (LH) and 4.1% and 3.0% (FSH). Analytical sensitivities were 0.5 IU/L (LH) and 0.05 IU/L (FSH).

**Figure 2.**
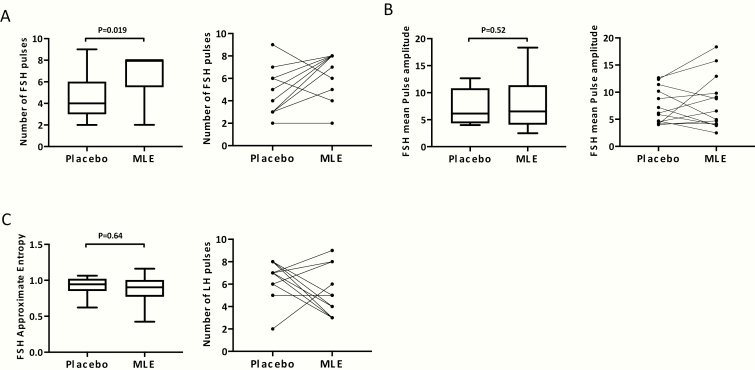
FSH pulsatility analysis when administered placebo or oral neurokinin 3 receptor antagonist [MLE4901] (MLE). FSH pulsatility was analyzed using a blinded deconvolution method with 93% sensitivity and specificity to calculate the number of FSH pulses, FSH pulse amplitude, and orderliness of FSH pulses (approximate entropy; the lower the number, the more ordered the pulses are, with zero denoting perfect orderliness). Statistical models implemented a standard crossover analysis, with administration sequence and treatment as fixed effects and subject as a random effect. (A) Left panel: number of FSH pulses; box plots: line, median; box, interquartile range; whiskers extend to the extremes of the data (minimum and maximum values). Right panel: individual values of number of FSH pulses. (B) Left panel: amplitude of FSH pulses; box plots: line, median; box, interquartile range; whiskers extend to the extremes of the data (minimum and maximum values). Right panel: individual values of amplitude of FSH pulses. (C) Left panel: orderliness of FSH pulses (approximate entropy); box plots: line, median; box, interquartile range; whiskers extend to the extremes of the data (minimum and maximum values). Right panel: individual values of orderliness of FSH pulses (approximate entropy).

No significant difference was found in mean serum FSH concentration when taking the oral NK3R antagonist compared to placebo (mean serum FSH: placebo 70.0 (standard deviations [SD] 19.6) IU/L, MLE4901 72.1 (SD 19.8) IU/L; p = .26 (see Figure 1A). The total number of FSH pulses increased (*P* = .032) with administration of the oral NK3R antagonist compared to placebo, but the amplitude and orderliness of pulses remained unchanged irrespective of treatment (*P* = .53 and *P* = .68 respectively) (see Figure 2).

However, mean LH concentration was significantly decreased when taking the oral NK3R antagonist compared to when taking placebo (mean serum LH: placebo 30.3 [SD 9.8] IU/L, MLE4901 27.6 [SD 9.76] IU/L; *P* = .0024) (see Figure 1B).

**Figure 3.**
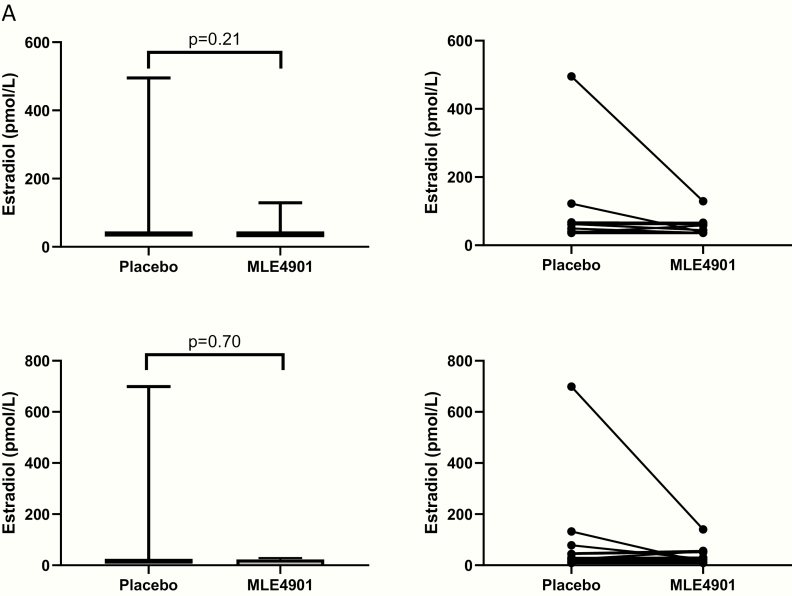
Left panel: mean serum estradiol concentrations during intervention periods calculated by immunoassay and highly sensitive direct assay: Serum estradiol was measured by 2 separate methods. The first, a priori, using a modern, commercially available, automated immunoassay (Abbott Diagnostics, Maidenhead, UK) (22). The second, post hoc, using a highly sensitive direct assay by LCMS. Mean concentration was calculated for the 4 weeks of the intervention periods (placebo and oral neurokinin 3 receptor antagonist [MLE4901]). Statistical analysis used Wilcoxon matched-pairs signed rank test as the data from the 27–28 participants who completed the protocol and consented to their remaining samples being used for post-hoc analysis. Tests were 2-tailed, significance was set at *P* < .05. (A) Left panel: mean serum estradiol concentration measured by immunoassay; box plots: line, median; box, interquartile range; whiskers extend to the extremes of the data (minimum and maximum values). Right panel: individual values of serum estradiol concentration measured by immunoassay. (B) Left panel: mean serum estradiol concentration measured by highly sensitive direct assay; box plots: line, median; box, interquartile range; whiskers extend to the extremes of the data (minimum and maximum values). Right panel: individual values of serum estradiol concentration measured by highly sensitive direct assay.

**Figure 4.**
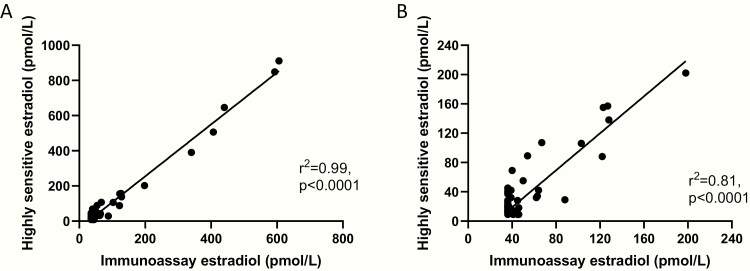
(A) Summary plot of serum estradiol concentrations during intervention periods as calculated by immunoassay and highly sensitive direct assays. Correlation coefficient *R*^2^ = 0.99 (*R*^2^ = 1 reflects a perfect linear association), therefore suggesting good concordance between the 2 analytical methods used. (B) Summary plot of serum estradiol concentrations ≤202 pmol/L during intervention periods as calculated by immunoassay and highly sensitive direct assays to account for the poor sensitivity of the immunoassay (included 160 of the total 165 paired results): Correlation coefficient *R*^2^ = 0.81, therefore suggesting good concordance between the 2 analytical methods used

Serum estradiol levels as measured by immunoassay were not significantly different when taking the oral NK3R antagonist compared to placebo (median serum immunoassay estradiol: placebo <37 pmol/L, MLE4901 <37 pmol/L; *P* = .21) (see Figure 3A). Furthermore, serum estradiol levels as measured by highly sensitive direct assay were also not significantly different when taking the oral NK3R antagonist compared to placebo (median serum highly sensitive estradiol: placebo 12 [interquartile range 16] pmol/L, MLE4901 13 [interquartile range 15] pmol/L; *P* = .70) (see Figure 3B). Good concordance was demonstrated between the two methods (modern, commercial immunoassay assay and highly sensitive direct assay by LCMS) in calculating the serum estradiol concentration; including when only values ≤202 pmol/L were included in analysis (160 of the total 165 paired samples) (see Figure 4).

## Discussion

In our a priori and post-hoc analyses, we have demonstrated that administration of an oral NK3R antagonist (MLE4901) to menopausal women does not change serum FSH or estradiol concentration, despite dramatic reductions in hot flashes.(11) This is particularly reassuring for women with a contraindication to conventional hormone therapy, such as those with a history of hormonally sensitive breast cancer or thromboembolism, those on estrogen deprivation therapy, and/or those at risk of deleterious bone loss. These results are consistent with the pre-existing data that has implicated NKB/NK3R signaling, via the median preoptic nucleus, as the mediator of menopausal hot flashes rather than a direct effect of estradiol per se and/or of LH pulsatility.(7, 9, 10, 24, 25) As good concordance was shown between the 2 assay methods used to measure serum estradiol and including when only lower values were analyzed in view of the poor sensitivity of the estradiol immunoassay, this suggests that the commercially available, automated immunoassay should be suitable to measure estradiol in these women because if the measured level is low then it is also likely to be low when measured using a direct, high-sensitivity assay. This is important for clinical practice as the automated immunoassay is widely available and cost effective unlike the high-sensitivity assay and because even low levels of estradiol have been shown to affect progression and risk for recurrence in women with hormonally receptive breast cancer. (15)

We also demonstrated that administration of MLE4901 reduced serum LH concentration. This observation is in keeping with data from our incident trial showing that the oral NK3R antagonist affects LH pulsatility by increasing LH pulse amplitude and orderliness but not number of pulses (11) as well as the work of others.(26, 27) However, while the total number of FSH pulses increased, FSH pulse amplitude and orderliness did not change with the administration of an oral NK3R antagonist compared to placebo, and this may in part explain why serum FSH concentration did not change. Such dissociation between LH and FSH levels has previously been demonstrated in individuals with hypogonadotropic hypogonadism secondary to mutations in TAC3 or TACR3 that encode NKB and NK3R, respectively.(28) Furthermore, while we demonstrated that circulating estradiol levels were unaltered, we are unable to exclude inhibition of local estradiol synthesis at the neuronal level.

Our study population was representative of women suffering with menopausal flashes as participants had a wide range of symptom duration, were ambulatory throughout the study without restrictions placed on their lifestyle, and included smokers; 74% were white. However, the number of participants was small, and the treatment duration was short. Thus, larger scale, longer term, studies will be informative.

The data that we report in this manuscript suggesting estradiol and FSH are unchanged as a consequence of oral administration of an NK3R antagonist despite improvement in hot flash symptoms is clinically relevant as there is significant interest in this drug class as a therapeutic for menopausal flashes (13) particularly in those in whom estrogen therapy is contraindicated. This finding provides reassurance that estradiol levels are not significantly altered during treatment with an oral NK3R antagonist and therefore may be practice-changing as it could give physicians the confidence to prescribe such agents to otherwise long-suffering women with a history of hormonally receptive breast cancer or those on estrogen deprivation therapy.

## Notes

***Clinical trial registration:*** The trial was registered in full at ClinicalTrials.gov prior to study start (NCT02668185).

## Additional Information

***Disclosure Summary:*** WSD is funded by the UK MRC and NIHR; was an inventor on a patent application 14762086.8-1453, which is registered to Imperial Innovations; was previously an investigator for a separate study of MLE4901 in polycystic ovarian syndrome, for which a consultancy fee was paid; and is a Consultant for KaNDy Therapeutics. Millendo Therapeutics provided some financial support for an administrative assistant for the study of MLE4901 in menopausal flashes. All other authors have nothing to declare. The views expressed are those of the authors and not necessarily those of the funders, the NHS, the NIHR, or the Department of Health.
